# Supplementary material for: Development and validation of a predictive nomogram for vascular crises in oral and maxillofacial cancer patients undergoing free flap surgery
Source: PLoS One. 2024 Dec 4;19(12):e0314676. doi: 10.1371/journal.pone.0314676 (PMC11616845; doi:10.1371/journal.pone.0314676)
Supplement: Attachment — Submitted filename: Response Letter.docx [file pone.0314676.s001.docx]

**Response letter**

**Subject**: Response to Edits Requested for Manuscript PONE-D-24-33122

Dear Editor，

I hope this message finds you well. Thank you for your feedback on our submission[PONE-D-24-33122]. Thank you for your thorough review and for the opportunity to improve our manuscript. We appreciate your valuable feedback.

Regarding your request to include the dates when the data were accessed for research purposes, we have made the necessary revisions. Specifically, we have now clearly indicated in the Methods section, on lines 116-117, that the data were accessed for research purposes from September 1, 2021, to November 30, 2021. We hope that this addition meets the journal’s requirements, and we apologize for any inconvenience caused by the initial omission.

Additionally, we appreciate you bringing the issue regarding the ethics approval letter to our attention. Upon reviewing the documents, we realized that the ethics approval covering the entire period of our study had already been obtained, but we inadvertently submitted the initial version that did not cover the full date range for participant recruitment and data collection. We have now attached the correct ethics approval letter, which ensures that our study was conducted within the approved ethical guidelines throughout the entire research process. We have also provided an English translation of this document for your convenience.

We sincerely apologize for any inconvenience these issues may have caused and appreciate your understanding and continued consideration of our manuscript.We hope these revisions address your concerns and comply with PLOS ONE’s policies and reporting standards.Please let us know if any additional information or clarification is required.

Thank you for your consideration.

Sincerely,

Xiaoqin Bi

West China Hospital of Stomatology,

Email: hxbxq@163.com
