# Supplementary material for: Development and validation of a predictive nomogram for vascular crises in oral and maxillofacial cancer patients undergoing free flap surgery
Source: PLoS One. 2024 Dec 4;19(12):e0314676. doi: 10.1371/journal.pone.0314676 (PMC11616845; doi:10.1371/journal.pone.0314676)
Supplement: Attachment — Submitted filename: Response to Reviewers.docx [file pone.0314676.s002.docx]

**Response Letter**

Dear Editor,

I am writing to submit the revised version of our manuscript titled "***Development and Validation of a Predictive Nomogram for Vascular Crises in Oral and Maxillofacial Cancer Patients Undergoing Free Flap Surger*y**" [PONE-D-24-33122]. We would like to express our gratitude to you and the reviewers for the thorough evaluation and insightful comments on our manuscript. We have carefully considered all the feedback and have made the necessary revisions to improve the quality of our paper. Please find below a detailed response to each of the reviewers' comments. The changes made in the manuscript are highlighted in the revised version for your convenience.

**Reviewer #1:**

**Comment 1**: [***Description of the cohort is incomplete. Data such as radiation therapy, type of free flap per patient should be provided***]

**Response:**

We appreciate the reviewer’s insightful feedback regarding the completeness of our cohort description. We acknowledge the importance of providing comprehensive data to enhance the clarity and robustness of our study.

- Radiation Therapy Data: This study was conducted in a tertiary dental specialty hospital (West China Hospital of Stomatology, Sichuan University), which primarily focuses on oral and maxillofacial surgery. Unlike general hospitals, this facility does not have an oncology department or provide radiation therapy and chemotherapy services.As a result, we could only assess whether patients had received radiation therapy prior to their surgical intervention. We recognize that this limitation may affect the overall understanding of the cohort.
- Type of Free Flap: In response to the request for information regarding the type of free flap per patient, we apologize for the initial oversight in our submission. We have now added this data to Table 1 and provided a detailed description of the various types of flaps used in the surgical procedures in Section 3.1, "Characteristic of Patients." (**Line 212-218**).We hope these additions will enhance the clarity and completeness of our study.

We appreciate the reviewers keen observation, and we will ensure that these points are clearly articulated in the revised manuscript to improve the overall quality of our study.

**Comment 2**: [***These data should be used in the comparison between training and validation cohorts]***

**Response**:

We appreciate the reviewers insightful suggestion regarding the comparison of data between the training and validation cohorts. As you pointed out, ensuring that the characteristics of both cohorts are well-matched is crucial for validating the robustness and generalizability of the predictive model. In our study, we have already compared the training and validation cohorts in terms of key demographic and clinical variables (**Line 205-207).** Specifically, we examined variables such as age, sex, comorbidities (e.g., hypertension, diabetes), tumor site, tissue flap details (type, size), and other perioperative factors. The results showed no significant differences between the two groups for most of these variables (Table 1). This demonstrates that the training and validation cohorts were well-matched, ensuring that the models performance was not influenced by imbalances between the two groups. Thank you for bringing this important point to our attention.

**Comment 3**: [ ***It isn't clear whether the biological data are pre or post operative ones, therefore if patients can be classified in "high risk" and "low risk" groups PRE operatively, which would be at greater value***]

**Response:**

Thank you for your valuable comments regarding the biological data classification. We would like to clarify the following points:

In our research, preoperative blood indicators were defined as the values measured on the second day after admission, prior to surgery. The postoperative blood indicators were measured within the first 24 hours post-surgery. For patients who developed vascular crises, the postoperative values represent the measurements taken before the onset of the vascular event. This method ensured that we captured important physiological changes both before and after surgery, enabling us to assess the relationship between these indicators and the occurrence of vascular crises.

While we did not explicitly divide patients into high-risk and low-risk groups, our study did analyze significant preoperative indicators (e.g., hemoglobin, D-dimer, albumin, and fibrinogen) that can help identify patients who are more likely to experience vascular complications. This provides an opportunity for clinicians to recognize potential risks before surgery and take preemptive actions, such as adjusting treatment plans or optimizing patient conditions, to potentially reduce the incidence of vascular crises.

By focusing on preoperative indicators, our findings contribute to early risk assessment and proactive management, enhancing patient care outcomes in clinical setting.

**Comment 4:** [***Association between vascular crisis and year of surgery is not clear : is it supposed to depend on surgeon's experience ?***]

**Response:**

We appreciate the reviewer’s insightful question regarding the association between vascular crisis and the year of surgery, particularly in relation to the surgeon's experience.

In our study, we included data from surgeries performed between 2014 and 2021. The decision to include the year of surgery as a variable in our analysis was motivated by two primary factors:

- To account for potential statistical differences across years: This was aimed at observing whether significant changes in outcomes occurred due to improvements in techniques or other temporal factors throughout the study period.
- To evaluate the influence of surgeon experience on the risk of vascular crises: We considered that increased experience over time might allow surgeons to refine their techniques, potentially reducing the occurrence of vascular crises.

However, our analysis revealed no statistically significant difference in the occurrence of vascular crises based on the year of surgery, suggesting that the year of surgery alone was not a strong predictor of vascular crisis. While trends indicated that patients' preoperative and postoperative hemodynamics became more stable over time, which correlated with a lower incidence of vascular crises, including the year of surgery in the model could hinder its generalizability. This is primarily because the level of surgeon experience and surgical techniques may vary significantly across different hospitals and regions.

As such, while surgeon experience could be a relevant factor, the year of surgery did not demonstrate sufficient statistical significance in our model. Including it may complicate the model's applicability and generalizability across different clinical environments.

Additionally, we have added Table 2(b) (**Line 538)**.To the manuscript to illustrate the results of the univariate analysis of classified data in the training group, further clarifying the relationship between different years and the incidence of vascular crises.

**Comment 5:** [***The nomogram, which is the major result of this sudy, has to be publicly available*** ]

**Response**:

We sincerely appreciate the reviewer's observation regarding the availability of the nomogram, which is a key outcome of this study. Due to an oversight during the submission process, we inadvertently failed to include the nomogram in our initial submission. We are grateful for the opportunity to address this issue. We have uploaded the nomogram, which can now be viewed in the attachments under the Figures section for public availability.

**Comment 6**: [***I think that a specific statistical reviewing is mandatory, to validate this intersting tool***]

**Response:**

We appreciate your insightful comment regarding the necessity of a specific statistical review to validate the nomogram presented in our study. We recognize that establishing the scientific validity of predictive tools is essential.

In our manuscript, we have thoroughly described the methodology used in developing the nomogram, including the statistical techniques employed for its construction and validation. We are confident that the rigorous approach taken in this study underpins the reliability and accuracy of the tool.

Thank you for your valuable feedback, which has prompted us to clarify the scientific basis of our nomogram in the revised manuscript.

**Journal Requirements:**

**Comment 1**: [***Manuscript Style Requirements***]

**Response:**

Thank you for your feedback. We have thoroughly reviewed the PLOS ONE style requirements and made the necessary adjustments to ensure that our manuscript adheres to the formatting guidelines. We appreciate your guidance in this process.

**Comment 2**: [***Please provide additional details regarding participant consent***]

**Response:**

Thank you for your thoughtful comments. In response to your request for additional details regarding participant consent, we have revised the Methods section(**Line 118-125**).

We have clarified that this study was retrospective, and all data were fully anonymized before being accessed by the research team. Additionally, we confirmed that none of the participants were minors, and written consent was not required due to the retrospective nature and anonymization of the data.

We hope these modifications address your concerns, and we appreciate your attention to this important aspect of the study.

**Comment 3**: [***Role of funder statement***]

**Response:**

Thank you for your inquiry regarding the role of the funders. We would like to clarify that the funders had no role in the study design, data collection and analysis, decision to publish, or preparation of the manuscrip. We have included the following statement in the Acknowledgments section of the manuscript **(Line 396-397**):

"The funders had no role in study design, data collection and analysis, decision to publish, or preparation of the manuscript."

Additionally, we have updated the cover letter to reflect this information. Please feel free to update the online submission form accordingly. Thank you for your understanding.

**Comment 4**: [***Data Availability Statement***]

**Response:**

Thank you for your request regarding the Data Availability Statement. We have updated the Data Availability Statement in the Manuscript (**Line 400-403**).To ensure it includes all necessary information. Please let us know if any further clarification is needed.

**Comment 5**: [***ORCID iD requirement***]

**Response:**

Thank you for the reminder regarding the ORCID iD requirement for the corresponding author. I have ensured that my ORCID iD is now validated in Editorial Manager as per your instructions. Please let me know if any further steps are required.

**Comment 6**: [***Please include a separate caption for each figure in your manuscript***]

**Response:**

Thank you for your suggestion. We have now included a separate caption for each figure , ensuring clarity and adherence to the journal's guidelines.

**Comment 7**: [***The autors should specify which package of R software they used***]

**Response:**

Thank you for your insightful comments. We appreciate your suggestion to clarify our data analysis methods. In our analysis, we utilized several R packages, each serving a specific purpose:

car: For statistical tests including t-tests, Wilcoxon rank-sum tests, chi-square tests, and Fisher’s exact tests.

survival: For survival data analysis using log-rank tests.

rms: For univariate and multivariate logistic regression and nomogram construction.

pROC: For generating ROC curves to assess model performance.

Hmisc: For data management and supplementary statistical functions.

rmda: For decision curve analysis to evaluate clinical utility.

We believe that the revisions have substantially improved the manuscript, and we hope that it now meets the standards of PLOS ONE. We are grateful for the opportunity to revise our work and look forward to your positive response.

Thank you once again for your valuable feedback.

Sincerely,

Xiaoqin Bi

West China Hospital of Stomatology,

Email: hxbxq@163.com
